# Experiences of foreign residents during COVID-19 pandemic in Taiwan

**DOI:** 10.1016/j.jmh.2022.100080

**Published:** 2022-01-21

**Authors:** Purnima Pandey, Mei-Kuei Yu

**Affiliations:** a Graduate Institute of Social Work, National Taiwan Normal University, Heping East Road, Section 1, Taipei, Taiwan; b Graduate Institute of Social Work, National Taiwan Normal University, SW508,5F,162 Heping East Road, Section 1, Taipei, Taiwan

**Keywords:** Life experiences, Community care, Cooperativeness, COVID-19, Taiwan

## Abstract

This study presents a survey analysis of the experiences of foreigners residing in Taiwan during the outbreak of the COVID-19 pandemic. Therein, we conducted interviews with 20 foreign residents of different nationalities in Taiwan. We shed light on participants’ expectations and experiences of living in Taiwan during a pandemic. As for analysis, we inductively analyzed various essential parameters, such as identifying categories, themes, and outlines, mainly from the surveyed data associated with the interviews. In this course, five relevant themes have appeared, namely (I) Access to proper care (II) Living environment (III) Psychosocial effects, (IV) Workplace Environment, (V) Impact of borders closure. The study concludes that foreign residents’ experiences during COVID-19 in Taiwan are admirable. The foreign residents feel comfortable, safe, and happy to stay and work in Taiwan because of the Taiwan government's policy towards successfully preventing community outbreaks.

## 1 Introduction

Taiwan is one of the safest countries in the entire world. It is only 1,010 km far from Wuhan, the epicenter of the COVID-19 virus, and the border of China is just 81 miles away. At the beginning of the COVID-19 pandemic, the virus was stated to be spreading worldwide (Gardner, 2020). However, the resident of Taiwan feels very safe. The main reasons include a state-of-the-art health care system, transparent information dissemination to the public, efficient production and transportation system, coordinated resource distribution, robust decision making for disease control measures, advanced technology, and collaborative public mindset (Wang et al., 2020). With the experience of SARS outbreak handling in 2004, the Taiwanese government was prompt to respond and started health inspection of arrivals from Wuhan on January 1 2020 (Chamarty, 2020). All the measures that the Taiwan government has been imposing have made all residents feel very safe. The government was serious and responsible. Similarly, every citizen took precautions and followed the guidelines issued by the government (Taiwan Centers for Disease Control 2020), such as wearing masks and maintaining safe distances in public places, elevators, and public transports. People were very aware and tried to avoid the spread of the virus.

So far, Taiwan has taken all the major steps to control the COVID-19 case. Till October 2021, the number of cases in Taiwan is 16309, and the number of death is 846 (Taiwan Centers for Disease Control 2020). The experiences and learnings from the 2003 SARS virus made Taiwan proactive in handling the COVID-19 (Lei and Klopack, 2020). Consequently, Taiwan has received worldwide recognition for its effort in restricting the virus at bay (Lin et al., 2020). Notice that it starts with the strict actions and implementation from the government, followed by the obedient Taiwanese citizens, residents, and businesses (Prevention and Control of COVID-19 in Taiwan).

Most countries around the world are in full lockdown mode. Globally, people are working from home, all major events have been canceled, and social distancing has become the new norm. It is still unclear when the world will come out of this pandemic, and even if we do, the world will be a whole new place. On the contrary, in Taiwan, it is an as usual day, businesses are operating like previously, kids are still going to schools, universities, offices, restaurants are still open. People in Taiwan live relatively routinely with proper protocols such as wearing masks and safe distancing measures at crowded places (Chiu et al., 2020). As early as January 2020, when many countries were still ensuring the potential danger of the virus, Taiwan proactively took action (Taiwan Centers for Disease Control 2020). The government began implementing strict and decisive measures that would inevitably help curb the spread of the virus within the nation. They ramped up the production of face masks and hospital equipment. Besides, in early January 2020, the Taiwan government stopped all the flights from Wuhan, China. Enacted travel restrictions and set up strict quarantine protocols for travelers coming from high-risk and affected countries (Taiwan Centers for Disease Control 2020). Surprisingly. in late December 2020, Taiwan had already informed WHO about the potential impact of the virus that emerged in Wuhan, China (Lin et al., 2020).

This study aims to examine qualitative descriptions of COVID-19-related experiences among foreigners living in Taiwan during the COVID-19 pandemic. Accordingly, we seek to address the following research questions:

1 What were the experiences of foreign residents in Taiwan during the COVID -19 pandemic?
2 How were the experiences of facilities offered to the foreigners by the government during the COVID -19 periods?
3 How did the public health measures affect foreign residents in Taiwan during the pandemic?

## 2 Methodology

### 2.1 Study design

The assumption of this qualitative study is based on representative inquiry that each individual (participants) perceives the world differently (Mantzoukas, 2004). This naturalistic method involves studying the spontaneous behavior of participants in natural surroundings (Salkind, 2010). This is done to acknowledge issues from their experience that could influence interpretation of participants stories (Lemon and Hayes, 2020). Further, the authors used thematic analysis of open-ended survey responses.

### 2.2 Data collection

Data were collected through separate interviews from December 2020 to February 2021. First, semi-structured in-depth interviews were conducted along with field notes were used as data collection strategies. Purposeful sampling continued until saturation, meaning no further data were obtained on the topics of interest (Naderifar et al., 2017). Interviews lasted between 25 to 60 min (mean 40 min). Interviews were transcribed verbatim. All the interviews were recorded with the participants’ permission and were transcribed before the qualitative analysis. To confirm privacy, each participant was assigned a pseudonym, and this was used, rather than their name, in the analysis and presentation of findings. All data were kept anonymous, and identifying data or information were removed as necessary. Foreigners were offered a choice of settings in which to be interviewed. In most cases, participants wanted the interviews to be conducted on their weekends. The interview was developed, covering open-ended questions to allow participants’ issues as they appeared. The interview was focused on the participants’ daily life in Taiwan during the pandemic and some specific questions s relating to their common support facilities provided by the government. For example, some of the questions asked were; 1) Do you know about Taiwan Covid-19 fighting Model?, 2) Are you satisfied with the government step taken for community transmission control facilities?, 3) As an immigrant, have you faced discrimination during these pandemic times?, 4) Is Covid-19 testing policy is covered in your National Health Insurance card(NHI)?, 5) Are you satisfied with the Taiwan government facilities during pandemic times for VISA extension or immigration issue?, 6) Overall, are you satisfied with Taiwan government facilities during pandemic times?, 7) Please give some positive and negative feedback for model doing work for the Covid-19 pandemic?, and among others.

The purpose of this study was to look into immigrants lived experiences and their coping strategic during pandemic times in Taiwan.

### 2.3 Data analysis

The transcribed data were analyzed thematically using a Framework analysis approach (Komori, 2019). Representative quotes were included in the results to illustrate the themes. The Framework approach was First established for realistic qualitative research. The thematic framework is used to categorize and form data which is specific to the study (Clarke and Braun, 2013). Following the conclusion of all interviews, each transcript was explored by observing applicable component and creating code with emerging themes and sub-themes highlighted, which aided the identification of themes that emerged through participants. In such a way that helped actual understanding and clarification of shapes, and as information was prepared according to case and theme (Goldsmith, 2021). The next level of analysis elaborates on the analysis of connections and relations between the themes. Researchers read the first transcripts independently and started a primary coding process distinctly to classify implications. These coding had been discussed and ordered into primary themes, and these themes were then used to produce an initial framework that had to guide the successive analysis (Nowell et al., 2017). The preliminary framework included all the coding and primary themes that were identified by the Authors.

### 2.4 Ethical consideration

Participants were informed about the ethical consideration, and participation consent was obtained from all the participants after the purpose of the study was explained to them using a participant information sheet. Furthermore, the research followed an ethical framework that entails voluntary participation, informed consent, the right to withdraw, anonymity, and confidentiality.

## 3 Results

### 3.1 Study of participants

The inclusion criteria for this study were that the immigrant participants should be 18 years of age and above, currently residing in Taiwan, able to speak English, and willing to participate in the study. The sample was chosen from various fields; (see Table 1). 9 were students; 1 was Engineer, 1 was Research Assistant, 6 were post-doctoral research fellows, 2 were Professors, and 1 was self-employed. The participants were interviewed individually. The participants were from the following countries: Indonesia, Thailand, Nepal, Vietnam, Bangladesh, Hong Kong, Mainland China, Turkey, Japan, USA, Mongolia, Iran, Philipines, Korea, Malaysia, Pakistan, and India. They were studying/working in different Universities/research institutes/companies. All participants volunteered to be in the study, and the information about the project was provided to participants beforehand. The individuality and continuity of the interviews not only facilitated the participants’ notion of the opportunity to refine their identity but also helped develop a deeper understanding of the immigrant people, as well as establish stronger bonding that facilitated telling stories of their experiences (Goldsmith, 2021). We have explored five themes: (I) Access to appropriate care (II) Living Environment (III) Psychosocial effects, (IV) Workplace Environment, (V) The impact of borders closure. In search of these categories, we find a variety of outcomes related to these daily life experiences in this pandemic situation.

**Table 1:** Participant details.

No	Country from	Gender	Age- Group	Years of living	Designation	Study/Work Place
1	Indonesia	F	20-30	1.5 Years	Student	National Yang-Ming University, Taipei, Taiwan
2	Indonesia	M	20- 30	6 Years	Student	National Taiwan University,Taipei, Taiwan
3	Thailand	M	20-30	6 months	Student	National Taiwan University,Taipei, Taiwan
4	Nepal	M	30-40	4 years	student	Academia Sinica, Research Institute, Nankang, Taiwan
5	Vietnam	M	30-40	3 Years	Student	National Central University, Taoyuan, Taiwan
6	Bangladesh	M	20-30	7 years	Student	Academia Sinica, Research Institute, Nankang, Taiwan
7	Pakistan	M	20-30	3 years	Student	Academia Sinica, Research Institute, Nankang, Taiwan
8	Mainland China	M	20-30	5.5 Years	Student	National Taiwan University, Taipei, Taiwan
9	Turkey	M	30-40	5 Years	Student	Academia Sinica, Research Institute, Nankang, Taiwan
10	Malaysia	F	30-40	11 years	Engineer	Science city Hsinchu, Taiwan
11	Hong Kong	M	20-30	3.5 Years	Research Assistant	Academia Sinica, Research Institute, Nankang, Taiwan
12	India	M	30-40	6 Years	Post-doctoral Fellow	National Taiwan University, Taipei, Taiwan
13	Japan	M	30-40	4 Months	Post-doctoral Fellow	National Taiwan University, Taipei, Taiwan
14	USA	M	30-40	3 Years	Post-doctoral Fellow	National Taiwan University, Taipei, Taiwan
15	Mongolia	M	30-40	4 month	Post-doctoral Fellow	National Taiwan University, Taipei, Taiwan
16	Iran	M	30-40	6 months	Post-doctoral Fellow	National Taiwan University, Taipei, Taiwan
17	Philippines	M	30-40	3 years	Post-doctoral Fellow	Academia Sinica, Research Institute, Nankang, Taiwan
18	India	F	40 Above	14 Years	Professor	National Taiwan University, Taipei, Taiwan
19	Korea	M	40 Above	10 Years	Professor	National Taiwan University, Taipei, Taiwan
20	Korea	M	40 Above	22 Years	Self Employed	Yonghe, New Taipei City, Taiwan

**Source:** Data from Field Work.

### 3.2 Access to appropriate care

The COVID-19 pandemic means that many of us stay at home and do less in terms of social interactions and exercise. This can have a negative effect on physical and mental health. However, in Taiwan, most of all activities have been running as usual (Wang et al., 2020). The Taiwan government established strategic and comprehensive medical facilities and emergency response plans for different community infection scenarios. To adequate medical manpower, the Taiwan government has extended the certificate renewal date for the entire healthcare workforce by six more months. Even extended the certificate renewal date for specialists and nurse practitioners by one more year (Prevention and Control of COVID-19 in Taiwan). Many expansions have been done for telemedicine. To expanding patient admissions, the government has correctly made plans for mild and severe patients. Also, negative-pressure quarantine wardrooms have been set up in quarantine hospitals and emergency response hospitals (Huang et al., 2020).

As one participant emphasized:

*For more precautions, in all hospitals, universities, and school health centers, visitors have to pass through two-step of screening. One at the fronts before entrance by the infrared thermal camera automatically scan temperature and sanitizer machine at entrance and 2nd one, manually by hospital workers. [P1]*

Before screening at the registration desk, a visitor needs to fill one form with questions about travel history, common illness, e.g., fever, cough, body pain, and respiratory symptoms. This series of checkpoint design lower the disease spread within the hospital. Similarly, at the airport, before immigration, people have to go through the same protocol (Huang et al., 2020). Alcohol for dry hand washing has been arranged at the entrance and exit, registration counter, Emergency Department, outpatient clinic, and other important locations (Prevention and Control of COVID-19 in Taiwan). Moreover, educational training and updates on the COVID-19 epidemic have been provided for the staff members based on the nature of their work (Chamarty, 2020).

As noted by one participant:

*If you have National Health Insurance card (NHI) than you can take easily all health facilities. During covid-19 pandemic times NHI card is working as an electronic card, and every pharmaceutical shop has this health card reader. [P14]*

With this heath card, the equal distribution of masks has been made available to everyone to avoid unnecessary panic buying. Further, to control the crowd at the mask shops, the government made the odd-even rule. In the odd-even rule, those who have an even number (last digit of NHI) can buy on Monday, Wednesday, and Friday, and for an odd number, one can buy on Tuesday, Thursday, and Saturday, respectively. However, after May 2020, the situation was mostly controlled, and there was no scarcity of masks.

Also, note that the Taiwan government launched its “Triple Stimulus Voucher” program to help stimulate Taiwan's sagging economy amid the COVID-19 pandemic (Kuo, 2021). The program allows Taiwanese citizens, as well as foreign and Chinese spouses, to purchase vouchers worth NT$3,000 (US$101) for the price of NT$1,000 (Kuo, 2021). However, this voucher was not for all foreigners. Basically, in Taiwan, there are two types of foreigners, one is permanent residency (called as Alien Permanent Resident Certificate (APRC) holder), and the other is not permanent (called as Alien Resident Certificate (ARC) holder). This Triple Stimulus Voucher was only for APRC holders.

As mentioned by one participant:

*As I and my family are Alien Permanent Residency (APRC) holders, Taiwan government have given “purchase voucher” of equivalent of approx. 101 USD for each family member, which effectively spurred consumption in Taiwan. These types of economy support its good chance of foreigners. [P18]*

The government's response is to decrease the possible negative impacts of the COVID-19 pandemic on the economy and society. The government had approved several special relief packages that included financial aid, employment assistance, and tax breaks.

During Covid-19, managing public transports was a big challenge for any government. Surprisingly, the Taiwan government did very well in this sector also. Taiwan public transports such as MRT, buses, and Trains run as usual with strict protocols (Chiu et al., 2020). Transport companies sanitize vehicles after every trip. People have to wear masks at all times when they use public transportation, and if a person refuses to wear a mask, they will be denied to enter or be fined up to NT$ 15000 (Chamarty, 2020). Also, there are temperature scanners and employees to monitor if everyone is wearing masks or not. The taxi company has stepped up efforts to contain the outbreak. Before their shift, all drivers have to monitor their body temperature and disinfected their vehicles, especially the place where passengers are likely to touch, such as door handle and seats (Taiwan Centers for Disease Control 2020). The driver has to wear a mask at all times while on shift. Such measures create a sense of safety in the mind of residents. The same condition follows for the bus services and metro services. Railway services have also strengthened epidemic control measures. They have been disinfecting the carriages and stations daily, and passengers were also required to have their temperature checked (Prevention and Control of COVID-19 in Taiwan). Most of the participants of this study appreciated Taiwan's transport facilities and safety measures. However, some participants mentioned that due to non-compliance to safer distancing measures during peak travel time, the frequency of buses and MRTs could have been increased.

### 3.3 Living environments

As already mentioned, most countries worldwide are in full lockdown mode with people working from home, all major events have been canceled, and social distancing has become the new norm (Inside Taiwan during COVID-19).

One of the participants stated that:

*When I first heard the news about a novel coronavirus in January, I was initially worried because Taiwan is right next to China. [P4]*

The government has prepared an improbable job defending this country against all odds. Taiwan on track applying protecting measures against COVID-19 since December last year. Healthcare professionals are working diligently on the front lines. Travel bans and strict quarantine measures were enacted early on. Tracking of coronavirus cases and contacts has been nothing short of impeccable (Wang et al., 2020). Mask production was ramped up, and a rationing system was implemented.

One of the participants said:

*Living in Taiwan during COVID-19 is…honestly, pretty normal. Taiwanese people take health very seriously. when they have a cough and cold, for instance, they always wear a mask in public places, including schools and public transportation. And they don't need the government to tell them to do that. They do it because they respect each other. [P6]*

The self-ruled island has one of the best records of virus containment in the world. On the other hand, here people follow the rule dutifully so here social distancing doesn’t really seem to be a main thing.

According to one of the participants:

*There are a few differences in day-to-day living since the outbreak, but nothing too drastic. There hasn't been any lockdown here at any stage like there has been in other countries for months, although it's been common practice to have your temperature taken whenever entering most shops, bars, restaurants, or offices*. [P5]

In Taiwan, people are still walking past each other no problem people are standing in line at each other or even like sitting on the train or the buses sitting next to strange and stuff like that because there are no such covid-19 cases reported from inside local people.

One of the participants said that:

*At our university, we are currently preparing for online learning in case that situation arises. We have some preventative practices that I hope schools in more countries will adopt and adapt. [P19]*

Taiwan also has protocols in place if there is a community spread in educational establishments. Taiwan erudite its lessons from SARS in 2003. The minimal changes to our daily lives in Taiwan are possible because the country implemented protective measures quickly, proactively, and effectively (Lei and Klopack, 2020). Culturally, living in a more collectivist as opposed to individualistic society also has a considerable impact. The normality of our current reality is a reflection of all of these factors and more. That being said, these are not reasons to relax just because we have fewer cases or a lower risk of community spread – flattening the curve does not work if not everyone participates or takes these measures seriously. We are just a really lucky foreigner to find myself living here, of all places (what-does-life-without-a-lockdown-look-like-776293). I hope the rest of the world learns their lesson from COVID-19 so that when a future epidemic occurs (because it certainly will), it does not have to turn into another devastating pandemic.

### 3.4 Psychosocial effects

The modern world in which all persons can travel and do social gatherings has been hardly forced to the present social isolation and limits. This unprecedented situation related to the COVID-19 outbreak clearly demonstrates that individuals are largely and emotionally unprepared for the detrimental effects of biological disasters that directly show how everyone may be frail and helpless. (Lei and Klopack, 2020). This is commonly one of the most psychological reactions to pandemics. Several existing studies demonstrated that those exposed to the risk of infection might develop pervasive fears about their health, worries about infecting others, and fear of infecting family members.

Some participants said:

*In Taiwan wearing a mask, it's kind of mitigates the need for the social distancing especially if you are not talking to each other, this is the preventive method. However, we don't feel upset or annoyed by this at all, actually we are like so grateful to be in Taiwan and just want to say thank you Taiwan. [P 6, 10 & 17]*

People are cooperative with the government in making all the investigations clearer and possible to the general public. Every day in every major newspaper there will be a doctor or some expert in an interview talking about some important issue of the disease.

Most of the informants stated that:

*Taiwan have encouraging life and campus alertness related to epidemic prevention; and very supportive with epidemic prevention efforts. Because all citizens are determined to fight the COVID-19 epidemic, their high levels of cooperativeness and voluntarily mask wearing have contributed to the successful epidemic prevention in Taiwan today.*

The transparency and supply of detailed information are very important in combating the pandemic. So far, Taiwan has been doing an outstanding job, and till October 2021, the number of cases here is 16309, and the number of death is 846 (Taiwan Centers for Disease Control 2020).

### 3.5 Workplace environment

During the COVID-19 period, in Taiwan, schools, colleges, offices, businesses, industries, public places, restaurants were open with certain restrictions. In all this place government asked them to arrange in such a way to avoid the crowd (Wang et al., 2020).

One of the participants said:

*In the seating place like in the canteen and restaurant, they have to put half the table right away They made a marker on the floor to stand on distancing for maintaining social distance. [P9]*

After all, in all places, hand sanitization machines and thermal scanners are compulsory at the entrance of every business, public gathering place, and restaurant buildings. Cleaners to make sure places like handrails or escalator handles are sanitized (Prevention and Control of COVID-19 in Taiwan). In education intuitions, such as universities, colleges, and research centers, they start to follow a proper rule for entry and stay on the campus. They put restrictions on other people.

One of the participants said:

*If you have a valid ID card, you can enter; if you don't have, you cannot. [P15]*

Educational institutions made such arrangements of scanning the ID card on the main gate of the campus and at the entrance of every department, canteen, office, and shop. Along with this restriction, they made a setup of thermal scans and automatic hand sanitizer at every place. In every educational institution, every day, all members have to go through a body temperature scan at the entrance of institutions and at the individual department (Prevention and Control of COVID-19 in Taiwan).

One of the participant share own experiences in working places:

*Mask made as compulsory through the campus for everyone. In-office and sitting places, they made the seating arrangement such that they follow the social distancing. In the university canteen and shopping place follow the same rule for entrance and seating.*[P11]

In universities, the maximum classroom seating arrangement follows the norm of social distancing. Also, some class teaching was running online by using zoom and other software. All areas were disinfected regularly. The government has utilized standard clean-up procedures in all public places. All schools (Kids, government, privates, and coaching centers) were open here during this pandemic. In school, kids go to school like a normal day. They have their daily temperature taken at the entrance gate and wear masks for the whole day (Inside Taiwan during COVID-19).

One of the participants, his kids who study in kindergarten, stated the situation of the school:

*They said first his kids hated to wear mask at first, but now they are habituated to use it and he wear whole day in class time. [P18]*

The classroom seating arrangement follows the norm of social distancing. Educational institutions and schools stop few things like sports center, swimming pool, annual function, and the conference for precaution during this period (Inside Taiwan during COVID-19). Moreover, one more important point is that many educational institutes at the beginning of February 2020 made a rule for every member that if anyone coming from abroad should not visit the campus for 14 days and should monitor their health condition every day and update it on the portal of institute health center website. During this period, pubs, clubs, and other prominent gathering places were closed by the government order. Department stores were less busy than usual day. In this pandemic, most of the businesses were affected. Therefore, the ministry of economic affairs and the ministry of finance come up with the package to help the industries in this situation (Prevention and Control of COVID-19 in Taiwan). There is no one-size-fits all clarification to the pandemic, public health legal awareness clarifies part of Taiwan's success.

### 3.6 The impact of borders closure

The Taiwan government has made border control policies stricter to prevent the disease from entering and spreading in Taiwan (Wang et al., 2020). Therefore, in the mid of December 2019, many reasonable steps were introduced concerning border control, such as thermal screening for fevers of arriving passengers, history of travel, occupation, contact, and cluster of suspicious cases (Lee, 2021). From mid of February 2020, the “Quarantine System for Entry” started online. The online system provides the incoming passenger with a portal to enter their health information before arrival, and the health declaration pass is automatically sent via SMS upon arrival in Taiwan (Wang et al., 2020). Also, with this system, the passengers can quickly clear customs by showing their SMS upon arrival; this improved the efficiency of border control. Gradually Taiwan government made the arrival rule stricter to stop the COVID-19 cases, such as, from March 19, 2020, all foreign travelers with exemptions to citizens and those holding a resident certificate or special entry permit were prohibited from entering Taiwan (Lee, 2021).

One of our participants told us:

*I went to our country for two weeks at the beginning of March and shortly after that I come back to Taipei. That time there had been a spike in the number of cases from overseas so the government changed the rules and said that anyone who returned to Taiwan had to immediately go into a 14-day quarantine. I notified the local authority, and they had called me and given some instructions related to quarantine. I was under strict instructions not to leave for two weeks, and they took my phone number and explained they would track my phone's GPS to ensure I did not break the rules. I was called twice or three times a day to check in and see if there was any change in my condition, and if I was okay for food and water. I even got a call on my last day thanking me for co-operating properly and apologizing for the inconvenience it had caused me even though it was actually the best two weeks of the year! [P18]*

One of the participants mentioned:

*Taiwan has kept tight border control of entry ports since then measures include symptom-based surveillance of everyone arriving by air and sea. [P13]*

On January 20 2020, Taiwan activated the Central Epidemic Command Center under its National Health Command Center. All the Ministries came together to formulate and implement policies. The Health Minister of Taiwan is heading this command center. All boundaries were sealed, and regular press briefings began. Taiwan's Digital Ministry used artificial intelligence to interconnect all the necessary data (Lee, 2020).

Besides, data was taken from insurance companies as to who has got insurance for travel abroad, and information was taken from the immigration Department. From February 18 2020, all the information started being given from the hospital to the clinic and the drug stores. So that the history of visits of every patient goes to everyone. Generally, governments use such information to control citizens, but Taiwan has gained the trust of its people in this matter (Wang et al., 2020). There the government can only do this at the time of disaster. Therefore, Taiwan is moving forward successfully to control this pandemic. Taiwan people follow all the rules implement by the government (Lee, 2020).

One of the participants said:

*Taiwan's airport is by far the most prepared airport on disease control. I'm just a little bit airsick so my temperature is higher than usual. Therefore, after arriving to Taiwan, Taiwan's disease control kept tracking me and calling me for a whole week, I'm not even from an Epidemic area. [P16]*

In this regard, many participants have appreciated these facilities, but some participants show unhappiness because of lots of unwanted official processes. However, a high level of trust with the government and public is the main reason Taiwan successfully contains the spread of COVID-19. That has allowed the island's economy to rebound, even though border controls are still in place (Chang et al., 2020).

## 4 Discussion

This study sheds light on the experiences of foreigners in Taiwan at the beginning of the COVID-19 pandemic. First, our findings revealed how these experiences were impacted by their multiple and intersecting identities as immigrants. The challenges of anguish aloneness and social loneliness offered by the pandemic, indicating flexibility and personal growth mainly with the acceptance of knowledge in their daily lives to keep on active. As the pandemic started in Taiwan, participants were exceptionally anxious. However, in Taiwan, foreigners are living their life in a normal way (Yeh and Cheng, 2020).

Participants had been reached out to cross-national support, primarily media via the internet and technology, including consuming news from both Taiwan and their country, transitioning to virtual activities via Zoom, and connecting with friends and family in both countries via WhatsApp or telephone (Lin et al., 2020).

However, these connections were not always positive. Many of our respondents were comfortable. However, effects of these experiences mixed among had been seen health-related anxiety was especially pronounced among our respondents (Yeh et al.,2019).

At the beginning of the pandemic, respondents, in particular, did not know where to seek help, and information from the authorities had not reached them because they had limited skills in the language of their host community and were unfamiliar with its official communication channels. However, the government had been broadcasting everyday situations by social media so that people would be aware and take more precautions in these pandemic times and help each other and support government precautions rule (Taiwan Centers for Disease Control, 2020). Due to good governance and clarity about COVID-19 cases, people feel better and know what to do so that this situation to be in control. In this region, even in the face of changing roles, foreigners were feeling comfortable. Overall, they were the most confident in the Taiwan authorities’ ability to manage the crisis and the most support of the Taiwan healthcare system. (Taiwan Centers for Disease Control, 2020).

Second, we highlight how were the facilities offered to foreigners by the government during the covid times in Taiwan, foreigners are still being invited to managerial necessities for status purposes, visa application, and renewal. However, granted flexibility on immigration requirements (e.g., automatic or simplified procedures for visa renewal or conversion, waiving fees) prevents widespread irregularity following the loss of employment and border closure migrants to come forward for health care and other assistance (Bureau of Consular Affairs, 2020). Third, in the context of facilities during pandemic times, ultimately, granting migrants regular status can drastically improve their access to health care and social security. The Central Epidemic Command Center required travelers arriving in Taiwan to home quarantine for 14 days. The Ministry of Foreign Affairs announced that all travelers who entered Taiwan on or before March 21, 2020, on a visitor visa, a landing visa, or through a visa-waiver program and who have not overstayed their legal stay period would be granted an automatic 30-day extension. A transparent approach to information management dealing with the pandemic. This method is applied in Taiwan, and it has been highly successful, particularly proven in the COVID-19 crisis in Taiwan (Cheng et al., 2020) . The government has taken many steps, and hence, people are enjoying life like a normal day in this COVID-19 pandemic (Yeh and Cheng, 2020).

### 4.1 Research limitations

Due to the characteristics of qualitative research, the sample size of this study was limited. First, most of the participants were doing work and study in university. Second, due to the nature of outbreak prevention, we were unable to conduct focus group interviews and did not collect data from various places in order to avoid potential cross-infection. In addition, this study was a short-term study. Long- term experience of the research subjects would be a valued possibility to explore in the future.

### 4.2 Suggestions

Foreigner residents recommended many services, strategies, and guidelines for their basic needs during their stay in Taiwan in pandemic times. Participants suggested that giving health care expenses if their relatives stay with them should also provide them with health care facilities during this pandemic. Government should provide some rebates in the new immigration policy when they come to Taiwan. They should provide allowance when they stay in quarantine center. Besides, the government should support special benefits for immigrants. For example, government financial support did not include immigrant residents. Some information on patients (like places where patients visited) was not shared with the public. Besides, the government should provide public announcements in English for non-Chinese speakers. The present model meritoriously controls the spread of the COVID-19 pandemic. However, some foreigners may have concerns about understanding the procedures displayed or spoken in Chinese in public areas. It will be better to provide the instructions in Chinese and English. This would allow the foreigners to comply with the government restrictions more effectively. In this pandemic situation, office time should not be restricted. Flexible working hours/ time by workplaces may reduce stressful situations during traffic (by MRT and buses). Online training and counseling support to workplaces can promote “work from home” culture to minimize the risk and spread of Covid-19 during traveling. The government can temporarily hire more people who deal with immigration and travel because many people in other countries take months to get a visa to Taiwan. Many of them lost their jobs during this time, and they could not manage very well due to the pandemic. Government can support such short-term and contractual employees. Government can open the gate for travel as long as visitors take the COVID-19 test and stay in the hotel for a 14-day quarantine. Moreover, the government needs to be more proactive in testing.

## 5 Conclusion

### 5.1 Concluding remarks

In conclusion, this research highlights the key aspects that have an essential effect on foreign residents’ experiences during the COVID-19 pandemic in Taiwan. In this study, emphasis is given to analyze how the government services control this pandemic situation to fight the disease efficiently. Taiwan's government utilized its community investigation system, big data from its immigration and national immigration digital databases, and mobile phone data for effective contact tracing, containment, and following of people in quarantine and isolation. Front-line health care workers could detect cases by accessing the integrated patient and travel histories. Eventually, we can say based on foreign residents’ feedback, Taiwan has phased this pandemic with transparency, accountability, and collaboration with the public, without lockdown and advanced preparation. So overall foreign residents are pleased with Taiwan's Covid-19 prevention strategy. They are very grateful to be here and to have been able to stay here. Taiwan has shown an example for other countries to follow on how to contain a pandemic successfully.

### 5.2 Future research

This study arranges a groundwork for future research in guidelines to address the needs of immigrants in modifying the effect of current COVID-19 and future global crises on their health and psychosocial well-being. Further, we could add numerical and traditional ethnographies and broader quantifiable and mixed-methods research that can produce a complete and more representative understanding of the impacts of the pandemic.

## Declaration of Competing Interest

The authors declare that they have no known competing financial interests or personal relationships that could have appeared to influence the work reported in this paper
